# Lumbosacral Vertebral Osteomyelitis With Iliopsoas and Epidural Abscess Following Intravesical Bacillus Calmette-Guérin Therapy

**DOI:** 10.7759/cureus.47421

**Published:** 2023-10-21

**Authors:** Anas M Abbas, Jenish Bhandari, Alex Ngan, Shaya Shahsavarani, Rohit B Verma

**Affiliations:** 1 Norton College of Medicine, Upstate University Hospital, Syracuse, USA; 2 Internal Medicine, Upstate University Hospital, Syracuse, USA; 3 Orthopedic Surgery, North Shore University Hospital-Long Island Jewish Medical Center, Manhasset, USA

**Keywords:** bacillus calmette-guérin, urology, spine, infectious disease, orthopedic, bladder cancer, bcg, osteomyelitis

## Abstract

Intravesical Bacillus Calmette-Guérin (BCG) therapy is the gold-standard adjuvant therapy for patients with superficial or non-muscle-invasive bladder cancer. BCG is a live attenuated strain of *Mycobacterium bovis*, which induces an antitumor environment, effectively fighting malignant uroepithelial cells through cytotoxic reactions. However, BCG therapy may stimulate local or disseminated infections. In rare cases, vertebral osteomyelitis may arise in the thoracolumbar spine, mostly affecting older males. This is a case of an 84-year-old male patient who developed L5-S1 osteomyelitis with associated epidural and iliopsoas abscess. Symptoms manifested as severe low back pain and bilateral lower extremity weakness. This paper aims to raise awareness of and educate spine surgeons in recognizing this uncommon complication by taking into context a history of BCG therapy.

## Introduction

Bacillus Calmette-Guérin (BCG) was initially developed in 1921 as a live attenuated strain of *Mycobacterium bovis* (*M. bovis*) and is currently used primarily as a vaccine against tuberculosis in children [1]. Beyond its use as a vaccine, BCG has been recommended as a form of intravesical immunotherapy against bladder carcinoma [1]. Morales et al. were the first to report the success of BCG in treating bladder cancer [2]. Over the past 40 years, BCG has been recognized as the gold-standard adjuvant therapy for superficial or non-muscle-invasive bladder cancer (NMIBC) [1].

The therapeutic effect of BCG is not well-understood as many proposed mechanisms exist. One mechanism of action is its direct cytotoxic effects on bladder cancer cell lines, thus inducing cell death or inhibiting cell proliferation [3]. A Th1 immune response mediated by CD8+ lymphocytes and natural killer cells supports an antitumor environment [3]. However, this immune reaction comes with a potential risk of side effects. Common side effects of BCG therapy are mild local and systemic reactions, including fever, rash, and urethral obstruction [4]. Serious adverse events are thought to occur in less than 5% of patients, including sepsis, osteomyelitis, or potentially death [5]. Vertebral osteomyelitis (VO) arising from BCG therapy is rare, occurring in less than 37 per 100,000 cases [6]. Although uncommon, VO is now recognized as a possible complication of this form of therapy for bladder cancer [7]. It is postulated that damaged uroepithelial cells spread to the spine through Batson’s plexus or from the seeding of the spine from disseminated infection [7]. Vertebral involvement may manifest with low back pain (LBP) initially but develop into motor weakness if not treated swiftly [7].

We present a rare case of BCG therapy prompting lumbosacral epidural and iliopsoas abscess with L5-S1 VO manifesting as LBP and bilateral (B/L) lower extremity (LE) motor weakness. This case is presented to bring awareness to this rare clinical occurrence that may mimic malignancy.

## Case presentation

An 84-year-old male presented to the emergency department with a three-month history of LBP and B/L LE weakness. The patient had a past medical history of chronic hepatitis C, essential hypertension, and bladder cancer, which was diagnosed one year ago. The patient had undergone three urologic procedures with spinal anesthesia and BCG therapy, which were well-tolerated until his last urologic procedure three months ago when the patient noticed B/L LE weakness thus mandating physiotherapy. Two weeks prior to admission to the emergency department, the patient noticed a significant increase in weakness in B/L LE and LBP, leaving him functionally immobile. LBP was described as excruciating, radiating bilaterally to the lateral aspect of his legs. The pain was intolerable and required him to crawl to bed and ambulate with a rolling walker. The patient was limited in performing activities of daily living, including turning over in bed, sitting down or standing up from a chair with the assistance of his arms, sitting up from bed, walking, or climbing more than three to five steps. These areas of deficit failed to resolve, despite treatment with heating pads, ice packs, physical therapy, and non-steroidal anti-inflammatory drugs. There was no history of trauma, orthopedic, or rheumatologic conditions. He had no history of drug allergy and did not display constitutional symptoms. The patient was transferred to our hospital for a spine surgery consult.

**Figure 1:**
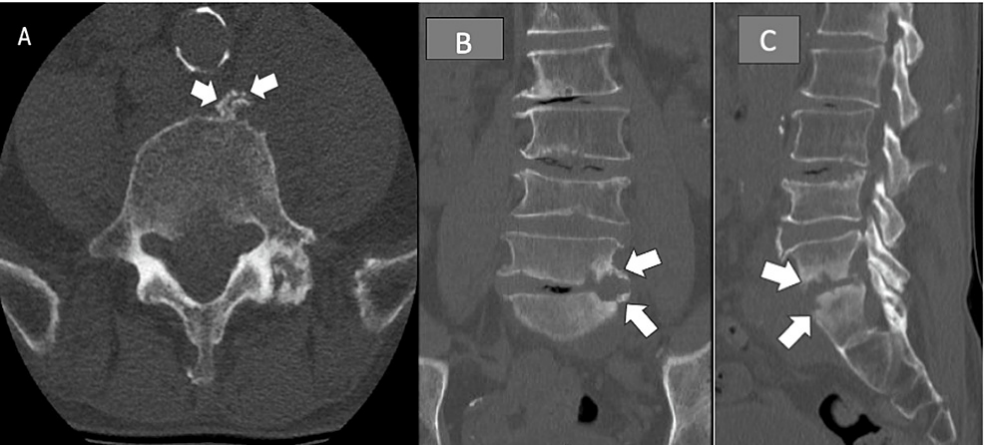
Preoperative multiplanar CT of the lumbar spine without contrast taken on the day of L5-S1 laminectomy for epidural evacuation. Axial (A) image shows the destructive process of the S1 vertebrae with an abscess in the left psoas muscle. Coronal (B) and sagittal (C) images show a deteriorated inferior endplate of L5 and a superior endplate of S1 (arrows).

The spine consultant was concerned about possible malignancy or tumor. He was afebrile with a normal white blood cell count but a C-reactive protein of 18 milligrams per liter (mg/L). The pain was reported as a 9 out of 10 on a verbal scale. Physical examination showed decreased range of motion (ROM) in B/L hip flexion and abduction limited by pain and weakness. LE strength was markedly decreased (B/L hip flexion, 2/5; B/L knee extension, 3+/5; B/L ankle dorsiflexion, 3+/5; B/L ankle plantarflexion, 2/5). Sensory deficits were found in the plantar surface of B/L feet. A computed tomography (CT) scan without contrast of the lumbar spine was ordered to rule out malignancy or tumors (Figure 1). The imaging showed abnormal areas of soft tissue mass along the left anterior aspect of the vertebral column effacing the fat plane between the vertebral column and the left psoas muscle. Epidural enhancements of the lower lumbar and upper sacral spine were concerning for abscess, osteomyelitis, or malignancy. An outside magnetic resonance imaging (MRI) scan of the lumbar spine showed an epidural abscess, which was impinging on the nerve roots. He started vancomycin and piperacillin/tazobactam. A spine surgeon performed an L5-S1 laminectomy for the evacuation of an epidural abscess. Epidural fluid was collected and sent to pathology for gram staining and culture.

The surgical pathology report noted a positive acid-fast bacillus (AFB) culture for a few rare acid-fast bacilli. Wound culture, anaerobic culture, and blood culture within the next four days were negative. Intraoperative findings were also concerning for spine tuberculosis and BCG abscesses. An infectious disease consult recommended beginning eight weeks of intensive anti-mycobacterial therapy (rifampin 600 mg daily; isoniazid 300 mg daily; ethambutol 1,200 mg daily), followed by seven months of isoniazid and rifampin therapy. The patient was placed on airborne precautions, which were later discontinued as AFB sputum returned negative twice. Prescribing oxycodone, Tylenol, and ketorolac controlled postoperative pain. The patient reported moderately improved pain following the procedure and from the pain medications, reporting the pain as a 6 out of 10. He started physical and occupational therapy. The patient was discharged six days after the procedure. Activities of daily living were performed independently but with mild difficulty, and the pain was controlled to a 4 out of 10. Physical examination showed 5/5 strength B/L LE with no sensory deficits and a slightly decreased ROM. The patient was recommended to continue the anti-mycobacterial therapy for eight months.

## Discussion

Tuberculosis is a granulomatous infectious disease caused by the gram-positive AFB under the genus *Mycobacterium*, which includes *M. tuberculosis*, *M. microti*, *M. africanum*, *M. bovis*, and *M. avium* [8]. Tuberculosis in humans is mostly caused by *M. tuberculosis* and is transmitted mainly through droplet infection [8]. *M. bovis* is the primary cause of tuberculosis infection in cattle but is also associated with human infection [8]. Humans are most likely to develop an *M. bovis* tuberculosis infection through the consumption of milk, milk products, or meat of an infected animal [8].

BCG was first introduced in 1921 and was accepted as a form of cancer treatment in 1969 when Mathé et al. reported the benefits of BCG in treating acute lymphoblastic leukemia [9]. The first report of successful BCG therapy in treating bladder cancer was reported in 1976 [2]. A proposed mechanism of action for BCG is initiated by binding fibronectin on damaged uroepithelial cells and then internalized into the cells [10]. This is followed by the expression of class II major histocompatibility complex in the cancerous cells, which becomes a target for lymphokine-activated killer cells and BCG antigen-presenting cells [10]. However, this reaction may provoke a granulomatous response with localized or disseminated infection [10]. Common local adverse events include cystitis and hematuria, which appear soon after treatment [10]. Systemic reactions include sepsis or potentially death, induced by a cytokine storm [10].

*M. tuberculosis* is the most frequent pathogen associated with VO [11]. In developed countries, the incidence of spinal infections from tuberculosis had decreased, with 6% involving VO [12]. Since the development of the BCG strain and its use in bladder cancer, VO has been a rare adverse effect, with a prevalence rate of 37 per 100,000 patients [6]. Compared to VO from *M. tuberculosis*, VO from *M. bovis* predominantly affects an older male population, most likely due to the higher incidence of bladder cancer associated with this demographic [11]. A review of 30 cases found that only males with a mean age of 73.4 developed VO from *M. bovis* [11]. *M. bovis* has been shown to promote VO in the thoracic and lumbar spine, with no reported cases involving the cervical spine [11]. Neurological complications and psoas abscesses were more frequent from BCG than an *M. tuberculosis* infection [11]. Lastly, *M. bovis* was not associated with other sites of infection with VO whereas *M. tuberculosis* was frequently associated with pulmonary tuberculosis with VO [11]. Our patient developed lumbosacral VO with a positive AFB stain for the *M. bovis* complex. Clinical findings, imaging, and the temporality of developing infection allowed us to rule out an *M. tuberculosis* infection in favor of a BCG-mediated response.

Psoas and epidural abscesses, as seen in Figure 1, are common sites of infection from BCG. Imaging in the form of MRI or CT found epidural abscess in 70% and psoas abscess in 47% of reported cases involving BCG therapy [13]. Although the iliopsoas abscess pathogenesis is not clear, it is accepted that BCG may spread from the bladder to the common iliac and paravertebral lymph nodes, which have a close anatomical relationship with the iliopsoas muscle [13]. At the iliopsoas muscle exists adjacent lymphatic drainage to the thoracolumbar column [13]. Another mechanism for BCG spread is through Batson’s plexus. Batson’s plexus fails to explain the spread of BCG to the thoracic spine through a venous system [7]. The anatomic difference between the two genders may explain the disproportionate prevalence of VO in males compared to females. Males have a vesical venous plexus, which may drain into the prostatic venous plexus, offering direct communication with the vertebral venous plexus [14]. Females lacking in the prostatic venous plexus cause BCG to follow a more circuitous pathway through the internal iliac veins to reach the vertebral bodies [14].

Clinically, the onset of symptoms from VO ranged from two weeks to 12 years following BCG instillation [14]. Weight loss and LE radiculopathies were common secondary complaints [14]. Back pain was found in 97% of the cases [11]. The median duration of back pain was five months and the median period of time between the last BCG instillation and onset of back pain was also five months [11]. Our patient reported LBP with lumbar radiculopathy down the lateral aspect of the legs and to the feet immediately following his last urologic procedure. The symptoms had resolved after three months following the L5-S1 laminectomy for epidural evacuation. It is possible that BCG may have spread to the patient’s spinal column prior to the third urologic procedure, only having manifested and exacerbated following the last treatment.

AFB staining is helpful in differentiating bacteria. Acid-fastness is an uncommon characteristic shared by the genera *Mycobacterium* and *Nocardia* [15]. *Mycobacterium* species do not absorb the cell wall with crystal violet very well and appear as light purple instead of the deep purple that is seen with other gram-positive bacteria [15]. The AFB staining from surgical pathology found a few rare AFB from the epidural fluid collection. However, blood cultures and peripheral cultures were negative for microorganisms. Negative culture results could be attributed to a false positive smear, but considering our patient’s history of BCG therapy, we believe that the AFB staining was a true positive for *M. bovis* bacilli. Furthermore, a false-positive stain would be unlikely considering imaging and intraoperative findings and concerns of abscess and VO. Irrespective of smear results, the mean time to detection (TTD) for all mycobacterium species is 15.3 days [16]. *M. bovis* only grew under one standard culture medium with a mean TTD of 11 days [16]. In our case, *M. bovis* was cultured for six days until the patient was discharged. This may not have been enough time to obtain a positive culture. Furthermore, the culture media used for *M. bovis* might not have been specific to nurture its growth.

Currently, there are no clear guidelines for treating VO associated with BCG therapy. Surgery should be a last resort, which means using pathogen-directed treatments as first-line agents. All strains of *M. bovis* and likewise BCG are universally pyrazinamide resistant so anti-mycobacterial therapy for a disseminated tuberculosis infection involves isoniazid with vitamin B6 supplementation, rifampin, and ethambutol for two months [17]. If the bacteria are susceptible to isoniazid and rifampin at the end of the intense period, ethambutol can be discontinued, and the patient should continue isoniazid and rifampin for nine months [17]. Subsequent BCG therapy should be halted [17]. The infectious disease team recommended undergoing intensive therapy for eight weeks with the three antibiotics followed by an additional seven months of consolidation therapy without ethambutol. Indications for surgery include progression of infection despite antimicrobial therapy, spine deformity or fracture causing excruciating pain, and neurologic compromise involving radiculopathy [14]. Our patient had an outside MRI, which showed an epidural abscess impinging on the lumbar nerve roots, most likely causing radiculopathy to the B/L LE. Because of the debilitating LBP and neurologic deficits, surgery was elected as the best course of action, despite the patient beginning intensive medication therapy. Laminectomy for epidural fluid evacuation had decompressed the patient’s nerve roots, providing immediate, albeit moderate, pain relief. While VO secondary to BCG has been reported yet limited, the majority of those patients were treated through intensive medication. Our patient developed an adverse event to BCG therapy, which required surgical intervention. To our knowledge, this case is one of very few that describes VO involving the sacral spine, as most cases of BCG therapy associated with VO involve the thoracolumbar spine.

## Conclusions

Intravesical BCG therapy is an effective treatment for NMIBC. However, patients must be educated on potential adverse events from this treatment. A live attenuated vaccine carries the risk of infection, and VO mimicking spinal malignancy should be recognized as a possible complication. Spine surgeons and oncologists should be aware of possible spinal infections in patients with a history of BCG therapy. However, surgery should only be indicated in patients with severe osteologic or neurologic conditions or in those who failed anti-microbial therapy.
